# Turning over New Leaves

**Published:** 1888-03-31

**Authors:** 


### TURNING OVER NEW LEAVES.

[Books for Review should be sent to The Editor, The Lodge, Porchester Square, W.]

### A Story of Doctors and Nurses.*

THE author of this decidedly clever novel seems to have written "The Massage Case" as a reminiscence of a very unpleasant personal experience. The story, briefly stated, is this: Elfie Campbell, the narrator, goes, when worn out with grief and the fatigue of nursing a dying mother, to live with an aunt, Mrs. Crawshay, whose lack of sympathy amounts to cruelty. Misunderstanding and harshness having reduced her to a state of extreme nervous exhaustion, Dr. Broadley, the physician consulted by Mrs. Crawshay on the subject, suggests that Miss Campbell should go to a nursing home, and there undergo a course of massage. The art, as the author describes it, consisted of "violent pinching from head to foot, and was a torture worthy only of the Spanish Inquisition." This, with the additional sufferings caused by electric shocks and the harshness of doctor, nurse, and landlady, each more unsympathetic than the other, reduces the patient to a state verging on insanity, from which she is rescued by Dr. Risedale, a physician whose skill in brain cases was due to his possessing the sympathetic imagination Dr. Broadley lacks. The nurse who works under him is equally unlike the *masseuse* from whom Elfie had previously suffered. She finally marries Dr. Risedale; but this, along with the complications wrought by an earlier love story, which allow of the introduction of a trip to Egypt, where the scenery is very well described and some opinions on politics are brought in, may be looked on as secondary to the main purpose of the book. That purpose is obviously to enter a protest, first against massage as a treatment for hysteria or any of the nervous diseases which are so often classed carelessly under that baneful name; and secondly, against doctors and nurses too readily believing the account of a patient's condition given by friends who, from lack either of affection or comprehension, may misrepresent it totally. From this point of view it is noteworthy for its studious moderation of tone. Not only have we the contrast between the two doctors and nurses, the good and the bad, but the good qualities of Dr. Broadley and the woman employed by him are honestly stated. There is no attempt to depict either as an impostor, and the doctor's energy and force of character are spoken of with frank admiration, although these are the main instruments in bringing the patient to the verge of madness. "From the outset," says the author, "he had sternly set his face alike against the humbug of the less conscientious of his colleagues and the importance of a class of patients to whom too much credence had been hitherto given. He had thereby made many enemies. But the number of his patients was legion, and he had been heard to remark that if a few of the most sensitive of them took fright and carried themselves and their ailments off to a more sympathetic physician, so much the better. He had enough to do without bothering himself about fussy people's aches and pains. After all, what is wanted in a doctor is not so much suavity of manner as a knowledge of his business. It could never be said of Dr. Broadley that, having listened to a patient's account of her ills, he contented himself with prescribing a harmless drug and pocketing his fee. He always made a sincere attempt at discovering the nature of the malady and relieving the sufferer." This is the good side of the man; the other is given in two sentences: "Start him right, and he would go right; but start him wrong, and you would with difficulty get him out of his groove. The great physician was extremely simple-minded, and could easily be misled by the thoughtless and unprincipled, provided nothing had occurred to put him on his guard." Such a character is perfectly real, perfectly possible; and while the mischief that results from his somewhat pachydermatous honesty and lack of fine perception is plainly stated—while we are shown that the very force and strength of character which had won him his place in the front of the profession tend to overawe his patient, and make her submit in silence to wrong judgment of her symptoms, there is no attempt to vilify Dr. Broadley himself, nor the profession to which he belongs.

The nurse is described with similar justice: "She was by no means a hard-hearted or ill-natured woman; only insufficiently trained and wanting in moral courage." Wanting in tact and insight also; for she strove to cheer her patient with harrowing tales of hospital experiences. Miss Benjamin, the landlady of the invalid's lodging-house, is more harshly treated. The discomfort and semi-starvation which Elfie Campbell enjoyed at a cost of eleven guineas a-week; the utter shamelessness of the landlady in her extortion and discourtesy; the callous way in which "the massage case" was made to feel that she was a "case" and not a sensitive human being in an exceptionally weak and excitable condition; all these things are shown up with stern clearness as well as with considerable humour. But the writer admits that Elfie's, though not an exceptional, is by no means a universal experience. He makes her state in the narrative of her sufferings that she discovered subsequently that she

* "The Massage Case." By Cyril Bennet. London: T. Fisher Unwin, 26, Paternoster Square. Two volumes.

could have had elsewhere peace, comfort, and proper attention, for about half what she paid at Miss Benjamin's "home"—as indeed we know from our own experience of the first home hospital, Fitzroy House, Fitzroy Square, W.

The accusations brought by a writer so evidently honest as the author of "The Massage Case" against this particular development of the medical or hospital system deserve to be treated seriously and examined fairly. Mr. Bennet evidently looks upon massage as more or less of a medical craze; but even then he admits that the case he describes can hardly be taken as a fair example of the treatment. "I will not blame the *Weir Mitchell* treatment for its failure to restore me to health. I have since carefully studied Dr. Weir Mitchell's able little book on the subject, and I have there found it stated that the patient should have an abundance of good food, that the nurse should be quiet and refined, that the massage should be painless, and that the doctor should win the confidence of the patient. In my case none of these conditions were complied with."

There are fashions in medicine as in all else, and massage is undeniably a fashionable medical treatment at the present time. It has its uses; if it were not in some degree beneficial, medical men of skill and repute would not recommend it in any case. But the cases for which it is suited are comparatively few. Where the patient desires and likes it, as she sometimes does, the moral effect is apt to be deleterious; she loses self-control and independence, and tends to degenerate into a languid and selfish invalidism, which is a disease in itself. For others, who are suffering from nervous disease and require rest above all things, it is simply cruel, besides being unnecessary. It is a variation of the theory that the best treatment for hysteria is a jug of cold water thrown over the patient. This may be advisable in hysteria itself, but is wickedly wrong in the mass of nervous ailments lumped together by the ignorant under that name. We have got over attempting the cure of madness by whips and chains; we must get over the idea that a delicate woman, suffering from over-strain or sorrow, which are present quite as often when she denies as when she admits their existence, is best treated with harshness.

The whole question of the duty of medical men in nervous cases is exceedingly difficult. A man may be an admirable doctor in all else, and yet diagnose these amiss. The symptoms affect the pulse but little, and the tongue not at all; to distinguish true illness from fretful self-indulgence taxes both the intuitions and the experience of the most skilful; to know when kindness is required and when severity, is almost beyond human skill. The patient will tell only half the truth, and her friends, whether sympathetic or the reverse, will misinterpret all they know; everything is so complicated, that we must not be too hard on the Dr. Broadleys, even when they blunder most. The only thing we can suggest for their guidance is, that they should cultivate keen observation, and a cautious and kind, we would even say reverent, spirit in their dealings with these difficult cases, and bear in mind a fact not always taught in the medical curriculum—that a human being has a soul as well as a body.

The duty of the nurse is a simpler matter to speak about. As our author says: "It is even more true of nurses than of other members of society, that a little knowledge is a dangerous thing."

The half-trained nurse, whether or not she holds a too easily gained certificate, is ten times worse than the uneducated woman whose place she has taken. The latter had generally drifted into her occupation through a helpful kindliness of disposition which brought her to the help of her humble neighbours before she was employed by the richer classes; and she had years of experience to make up for the lack of methodical training. She was incomparably inferior to a woman possessed of similar natural gifts who has been trained to that scrupulous attention to every detail of a nurse's duties which is learned in our best hospitals; but she is better than those women, naturally cold and often careless, who take up nursing as a trade, and on the strength of an acquaintance with some tricks and terms, picked up in a month or two spent in a hospital ward, pose before the world as trained nurses. The training which makes a nurse less of a woman, and puts automatic accuracy in the place of human sympathy is not an unmingled good; and if this book, which, under the guise of a story, points out clearly, but with a not unfriendly hand, the errors into which both branches of the medical profession are apt to fall, makes doctors and nurses more careful and kind, we, at least, will bid it welcome. "To see ourselves as others see us" may not always be a pleasant experience, nevertheless it may be a wholesome one.

### Bournemouth and its Surroundings.*

A LARGE number of people among our hard-working and change-seeking town populations feel a keen interest in the subject of health resorts. It really is a matter of very considerable moment when a man has been six weeks in bed with a dangerous illness, and can with difficulty spare even two more for change and convalescence, that he should be able to choose with some certainty a seaside place which will rapidly bring back the blood to his cheeks and the courage to his flagging heart. Although the health resorts of Great Britain are numerous, varied, and possessed of excellent restorative qualities, it is not always easy to choose exactly the right place at the right time. It is not everyone who has an *Encyclopædia Britannica* on his bookshelves, nor is every library furnished with a separate volume containing a full, true, and particular account of every separate sanatorium in the kingdom. We are glad, therefore, when any single "place of health" finds its own proper scribe. Bournemouth may surely be congratulated on having found a judicious exponent of its many climatic and geographical merits in Dr. Dobell. The book before us is too long for any adequate description of its contents to be given in the space at our disposal. This much, however, is due to be said—that it is a second edition; that it is easily and pleasantly written; that it gives a vast amount of valuable and interesting information, not only about Bournemouth, but about many other places which the writer's extensive experience and excellent powers of observation have made him familiar with; that it is not unduly laudatory of its subject, but endeavours to be, and succeeds in being, judicial and reliable. Bournemouth is undoubtedly one of the healthiest and pleasantest of English health resorts, and anyone who wishes to be well informed of its general and particular merits cannot do better than consult Dr. Dobell's lively and instructive pages.

### Organic Materia Medica.†

MR. BENTLEY has placed medical and pharmaceutical students under an obligation by the publication of this little work. It is brief clear, systematic, and correct. The different officinal plants are given, with their natural history, modes of preparation, and medicinal uses. The book does not profess to teach the science and art of therapeutics; but as an introduction to therapeutics it will be found valuable. By those who are preparing for examination, and who are compelled to read text-books in which many other matters are mixed up, it will be specially appreciated. It may be read through in a day or two, and its price is only seven and sixpence.

---

BOOKS received: *The Church of England Temperance Chronicle:* T. B. Burrow, 7, Paternoster Square. *The International Record of Charities and Correction:* G. P. Putnam's Sons, New York and London. *The Provincial Medical Journal. Nasal Polypi*, Woakes. London: H. K. Lewis, Gower Street. *Prosperity and Pauperism:* Edited by the Earl of Meath. London: Longmans and Co. *The Drapers' Record.* London: McRae, Curtice and Co., Catherine Street, Strand, W.C. *The Empress:* T. Black and Co., Calcutta.

---

*"Bournemouth and its Surroundings." By Horace Dobell, M.D. London: Smith, Elder, and Co.

† "Organic Materia Medica." By Robert Bentley, M.R.C.S., F.L.S. London: Longmans, Green, and Co.

